# Phospho-ablation of cardiac sodium channel Na_v_1.5 mitigates susceptibility to atrial fibrillation and improves glucose homeostasis under conditions of diet-induced obesity

**DOI:** 10.1038/s41366-021-00742-4

**Published:** 2021-01-26

**Authors:** Revati S. Dewal, Amara Greer-Short, Cemantha Lane, Shinsuke Nirengi, Pedro Acosta Manzano, Diego Hernández-Saavedra, Katherine R. Wright, Drew Nassal, Lisa A. Baer, Peter J. Mohler, Thomas J. Hund, Kristin I. Stanford

**Affiliations:** 1 Department of Physiology and Cell Biology, The Ohio State University Wexner Medical Center, Columbus, OH USA; 2 Center for Diabetes and Metabolism Research Center, Dorothy M. Davis Heart and Lung Research Institute, The Ohio State University Wexner Medical Center, Columbus, OH USA; 3 Department of Biomedical Engineering, The Ohio State University, Columbus, OH USA; 4 Frick Center for Heart Failure and Arrhythmia, Dorothy M. Davis Heart and Lung Research Institute, The Ohio State University Wexner Medical Center, Columbus, OH USA; 5 Department of Internal Medicine, The Ohio State University Wexner Medical Center, Columbus, OH USA

**Keywords:** Cardiovascular biology, Metabolism

## Abstract

**Background:**

Atrial fibrillation (AF) is the most common sustained arrhythmia, with growing evidence identifying obesity as an important risk factor for the development of AF. Although defective atrial myocyte excitability due to stress-induced remodeling of ion channels is commonly observed in the setting of AF, little is known about the mechanistic link between obesity and AF. Recent studies have identified increased cardiac late sodium current (*I*_Na,L_) downstream of calmodulin-dependent kinase II (CaMKII) activation as an important driver of AF susceptibility.

**Methods:**

Here, we investigated a possible role for CaMKII-dependent *I*_Na,L_ in obesity-induced AF using wild-type (WT) and whole-body knock-in mice that ablates phosphorylation of the Na_v_1.5 sodium channel and prevents augmentation of the late sodium current (S571A; SA mice).

**Results:**

A high-fat diet (HFD) increased susceptibility to arrhythmias in WT mice, while SA mice were protected from this effect. Unexpectedly, SA mice had improved glucose homeostasis and decreased body weight compared to WT mice. However, SA mice also had reduced food consumption compared to WT mice. Controlling for food consumption through pair feeding of WT and SA mice abrogated differences in weight gain and AF inducibility, but not atrial fibrosis, premature atrial contractions or metabolic capacity.

**Conclusions:**

These data demonstrate a novel role for CaMKII-dependent regulation of Na_v_1.5 in mediating susceptibility to arrhythmias and whole-body metabolism under conditions of diet-induced obesity.

## Introduction

Atrial fibrillation (AF) affects ~3 million people in the USA alone [1] with an expected incidence of 15 million by 2050 [2]. AF is highly correlated with multiple risk factors including heart failure, age, obesity, and type 2 diabetes (https://www.cdc.gov/obesity/adult/causes.html) [3–5]. Among these, the growing incidence in obesity (https://www.who.int/en/news-room/fact-sheets/detail/obesity-and-overweight) has been identified as a major risk factor for AF [1, 6, 7] and is implicated in 17.9% of all AF cases [7]. Although the incidence of AF and obesity is rapidly increasing worldwide, the causative link between the two pathologies is not clear.

It is well-established that atrial excitability undergoes dramatic changes in the setting of AF, which further exacerbates the substrate for atrial arrhythmia (atrial remodeling). Studies have shown that defects in atrial myocyte Ca^2+^ handling are important in atrial remodeling, leading to aberrant membrane excitability and dysregulation of critical Ca^2+^-dependent signaling pathways [8, 9]. Previous work from our group and others showed that calmodulin protein kinase II (CaMKII) dependent regulation of voltage-gated sodium channels (Na_v_) is a critical determinant of AF susceptibility in animal and humans [10–16]. Specifically, CaMKII phosphorylates the alpha subunit of cardiac Na_v_ (Na_v_1.5) at Ser571 in the DI-DII linker, leading to increased pathogenic late current (*I*_Na,L_), which promotes arrhythmogenic action potential after depolarizations and further disrupts intracellular Ca^2+^ handling [8–10, 15]. This increase in *I*_Na,L_ has been observed in animal models and patients with AF, and drugs that target *I*_Na,L_ have shown promise as therapeutic targets for AF. Importantly, whole-body knock-in mice lacking the Ser571 site in Na_v_1.5 (SA mice) have reduced AF inducibility [8].

Obesity increases pericardial fat mass, induces left atrial remodeling, and is a major risk factor for AF [17–21]. Although *I*_Na,L_ has been recognized as an important driver of AF, the impact of obesity on *I*_Na,L_ has not been studied. Here, we investigated whether diet-induced obesity increased susceptibility to AF in wild-type (WT) mice, and whether the SA knock-in mouse model had reduced susceptibility to AF, even in the presence of a high-fat diet. Similar to previous studies [22, 23], we found that diet-induced obesity increased AF susceptibility compared to age-matched, chow-fed WT mice. Ablation of CaMKII-dependent phosphorylation of Na_v_1.5 was protective against the development of AF under conditions of diet-induced obesity. Surprisingly, we also found that the SA allele improved glucose metabolism, and reduced body weight gain associated with HFD, likely due to decreased food intake. Mitigating the difference in food intake by pair-feeding WT mice to SA mice reduced atrial arrhythmia events in WT mice, but the improvements in glucose metabolism were still maintained. Taken together, these data highlight the novel role of Na_v_1.5 in mediating susceptibility to AF and whole-body glucose metabolism under conditions of obesity.

## Methods

### Animals

Male, 18–24-week-old C57BL/6 mice from Jackson Laboratory (WT mice) or *Scn5a* knock-in mice with a Ser-to-Ala mutation at Ser571 of the cardiac voltage-gated channel Na_v_1.5 (SA mice) were used for all experiments [10]. Animals were housed at room temperature (22 °C) on a 12-h light/dark cycle. All procedures were conducted in accordance with the Guide for the Care and Use of Laboratory Animals published by the National Institutes of Health following protocols approved by the IACUC at The Ohio State University. Animals were euthanized using isoflurane and cervical dislocation followed by collection of tissue or cell isolation.

### High-fat diet and pair feeding

WT and SA mice were fed a chow (20% kcal from fat; Teklad) or high-fat diet (60% kcal from fat; Research Diets Inc.) for 6 or 12 weeks. WT and SA mice were fed *ad libitum* throughout the study unless otherwise indicated. In a subset of experiments, pair-feeding of WT and SA mice was performed by measuring daily food intake of SA mice and calculating the difference in food consumption from the previous day. Each WT mouse was randomly paired to a specific SA mouse and fed the calculated food consumption for that SA mouse each day for 6 weeks.

### Atrial arrhythmia susceptibility

To measure susceptibility to atrial arrhythmia events in vivo, subsurface ECGs were obtained from anesthetized mice using subcutaneous ECG leads in the lead II configuration and recording software (Powerlab, ADInstruments). Mice were anesthetized using 2% isoflurane with an oxygen flow rate of 1.0 L/min, and then placed in laying position on a heating pad to maintain body temperature. 1.5% isoflurane was used to maintain anesthesia. Recordings were taken at baseline for 3 min, with epinephrine (1.5 mg/kg, intraperitoneal) for 3 min, and with caffeine (120 mg/kg, intraperitoneal) for 10 min. LabChart (ADInstruments) was used to analyze the data for premature atrial contractions (PACs) in the 10 min following epi/caffeine injection (PACs/10 min), as well as for the severity of atrial tachycardia or fibrillation (AT/AF). AT was defined as at least three premature *p*-waves in rapid succession (i.e., three premature p-waves within 100 ms), while AF was defined as a period of R–R variability without discernible *p*-waves. The AT/AF score was assigned based on the duration of AT/AF during the 10 min following epi/caffeine injection, with a score of 0 corresponding to no incidence of AT/AF, a score of 1 to <1 s of AT/AF, a score of 2 to 1–10 s of AT/AF, a score of 3 to 10–60 s of AT/AF, and a score of 4 to >1 min of AT/AF. A subset of WT mice fed a high-fat diet was injected with mexiletine (25 mg/kg) or saline 15 min before baseline ECG recording began.

### Echocardiography

To assess left atrial remodeling, echocardiographic images were obtained from anesthetized mice following 6 weeks of HFD. Mice were anesthetized using 2% isoflurane, and 1.5% isoflurane was used to maintain anesthesia. Left atrial diameter was measured along the parasternal long axis view using a Vevo2100 (VisualSonics) system with the MS-400 transducer.

### Histology and imaging

Left atria were fixed in neutral buffered 10% formalin, processed routinely into paraffin, and then sectioned serially at 5 µm. Sections were stained using Masson’s Trichrome to examine the amount of interstitial and perivascular fibrosis, wheat germ agglutinin Alexa488 Conjugate to evaluate cell cross-sectional area, and TUNEL kits to examine the amount of apoptosis. Fibrosis was quantified using a custom-built MATLAB program [24]. Cell cross-sectional area was evaluated using ImageJ.

### Body composition and metabolic testing

Body weight was measured using an OHAUS NV212 scale. Body fat and lean mass were measured using an EchoMRI instrument (EchoMRI LLC) with canola oil calibration [25]. Glucose tolerance testing was performed after a 12-h fast with drinking water available *ad libitum*. Blood glucose was assessed at baseline by a tail vein prick. Glucose was administered by intraperitoneal injection (2 g glucose/kg body weight or per kg lean mass) at 0 min, and the tail vein prick was used to measure blood glucose levels at 15, 30, 60, and 120 min post injection [25]. Insulin tolerance testing was performed following a 2 h fast with drinking water *ad lib*. Baseline blood glucose levels were measured using a tail vein prick. Insulin was administered by intraperitoneal injection (1 unit per kg body weight) at 0 min. Blood glucose levels were measured at 10, 15, 30, 45, and 60 min post injection. If at any time a mouse dropped below 40 mg/dL glucose, they were given an intraperitoneal injection of 200 μL of 20% glucose (0.1 g/mL) and subsequently removed from the test. Pyruvate tolerance testing was performed after a 12-h fast with drinking water available *ad libitum*. Baseline blood glucose levels were measured using a tail vein prick. Pyruvate was administered intraperitoneally (2 g sodium pyruvate/kg body weight) at 0 min and blood glucose levels were measured 15, 30, 45, 60, and 90 min post injection [26].

### Comprehensive lab animal monitoring system

The Comprehensive Lab Animal Monitoring System (Oxymax Opto-M3; Columbus Instruments) was used to measure activity level, volume of O_2_ consumption, volume of CO_2_ production, and heat production. Total energy expenditure of mice was calculated as described previously [27]. Data were collected over 48 h; 24 h in the fed state and 24 h in the fasted state.

### Quantitative PCR

Tissue processing and quantitative PCR (qPCR) were performed as previously described [28]. Sigma-Aldrich custom primers were used for genes of interest with the sequences shown in Supplementary Table 1. All qPCR gene expression was normalized to the housekeeping gene GAPDH.

### Western blotting

Tissue processing and immunoblotting were performed as previously described [26]. The GAPDH (Fitzgerald; 10R-G109A), phosphorylated CaMKII at Thr286/287 (pCaMKII) (Thermo Fisher; MA1-047), and CaMKII (Badrilla; A010-56AP) antibodies were commercially sourced, and phosphorylated Na_v_1.5 at Ser571 (pNa_v_1.5) and Na_v_1.5 antibodies were custom generated, as described previously [10, 15]. All immunoblotting data were normalized to GAPDH.

### Statistical analysis

GraphPad Prism 7 (GraphPad Software, San Diego, CA, USA) was used for statistical analysis. The sample sizes in each experiment are provided in figure legends. Only ill and/or wounded animals were excluded from the analyses (*n* = 2 in this study). The data are presented as means ± SEM. Statistical significance was defined as *P* < 0.05 and determined by two-tailed *t*-test or one- or two-way ANOVA, with Tukey and Bonferroni post hoc analysis.

## Results

### SA mice are resistant to weight gain and atrial arrhythmias induced by high-fat diet

**Fig. 1:**
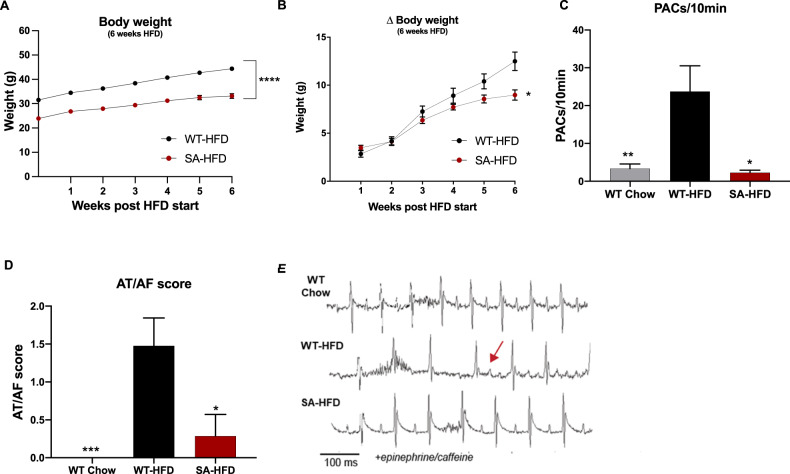
SA knock-in allele confers resistance to body weight gain and reduces susceptibility to atrial fibrillation on a high-fat diet. **A** Body weight of WT-HFD and SA-HFD mice after 6 weeks of HFD; **B** changes in body weight for WT-HFD and SA-HFD mice after 6 weeks of HFD. Data are presented as means ± SEM (WT-HFD *n* = 27, SA-HFD *n* = 13; *p < 0.05, ****p < 0.0001 vs WT-HFD). **C** Density of pre-atrial contractions; **D** severity of AT/AF based on a score of 0 (none) to 4 (severe); and **E** representative ECGs following injection of epinephrine (1.5 mg/kg) and caffeine (120 mg/kg). Data are presented as means ± SEM (WT Chow *n* = 22, WT-HFD *n* = 23, SA-HFD *n* = 14; *p < 0.05, **p < 0.01, ***p < 0.001 vs WT-HFD).

To investigate the role of diet-induced obesity on susceptibility to AF, WT and SA mice were fed a high-fat diet for 6 weeks (WT-HFD and SA-HFD, respectively). SA-HFD mice had reduced total body weight and body weight gain compared to WT-HFD mice (Fig. 1A, B).

To determine the effects of HFD on susceptibility to atrial arrhythmia, WT-HFD mice, age-matched chow-fed WT (WT Chow) mice, and SA-HFD mice underwent adrenergic challenge using epi/caffeine, and changes in the incidence of atrial arrhythmia events [PACs and atrial tachycardia/fibrillation (AT/AF)] were measured. WT-HFD mice had increased incidence and severity of PACs and AT/AF compared to WT Chow mice (Fig. 1C–E). Interestingly, SA-HFD mice were resistant to the HFD-induced increase in atrial arrhythmia events (Fig. 1C–E). Similar to their response on a chow diet [8], SA mice had little to no PACs or AT/AF events even after 6 weeks of a high-fat diet. Consistent with a role for CaMKII-dependent phosphorylation of Na_v_1.5 in atrial arrhythmia, elevated levels of phosphorylated/activated CaMKII (pCaMKII) and phosphorylated Na_v_1.5 at S571 (pNa_v_1.5) were observed in whole heart lysates from WT-HFD compared to WT Chow without any difference in total Na_v_1.5 (Sup. Figure 1A). Interestingly, SA-HFD hearts were resistant to the increase in pCaMKII observed in WT-HFD (Supplementary Fig. 1B) (pNa_v_1.5 was not evaluated in SA-HFD due to the loss of antibody epitope as previously reported [10]). Overall, these data indicate that preventing CaMKII-dependent phosphorylation of Na_v_1.5 confers resistance to body weight gain and reduces susceptibility to AF under conditions of diet-induced obesity.

### Inhibition of the late sodium current with Mexiletine reduces susceptibility to arrhythmias

**Fig. 2:**
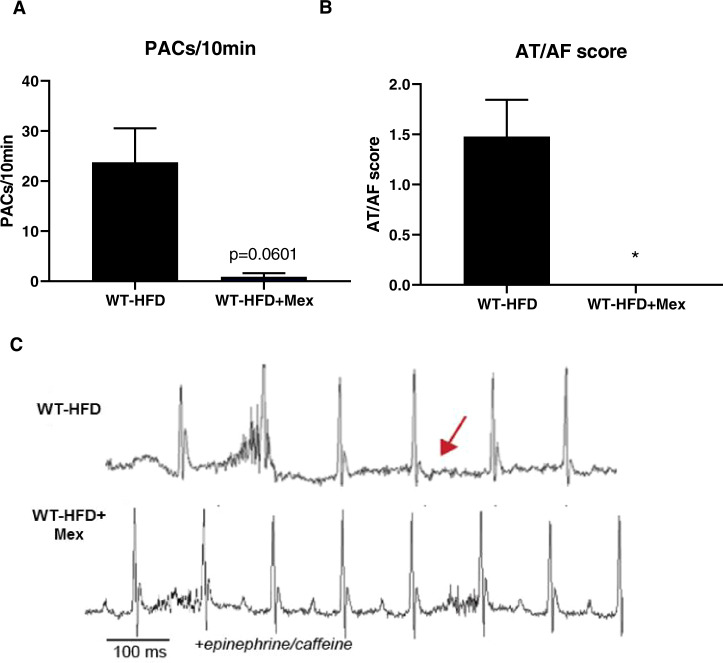
Inhibition of *I*_Na,L_ with Mexiletine reduces susceptibility to atrial fibrillation under conditions of diet-induced obesity. **A** Density of pre-atrial contractions; **B** severity of AT/AF based on a score of 0 (none) to 4 (severe) following injection of epinephrine (1.5 mg/kg) and caffeine (120 mg/kg); and **C** representative EKGs from 6-week high-fat diet mice injected with saline or mexiletine followed by exposure to epinephrine/caffeine. Data are presented as means ± SEM (WT-HFD *n* = 23, WT + HFD + Mex *n* = 8) (*p < 0.05 vs WT-HFD).

Given that increased *I*_Na,L_ leads to atrial arrhythmias in high-fat fed mice, we assessed the effect of the Na^+^ channel blocker mexiletine on arrhythmia susceptibility in WT-HFD mice [8]. Consistent with the hypothesis that increased *I*_Na,L_ downstream of CaMKII-dependent phosphorylation of Na_v_1.5 increases susceptibility to obesity-induced AF, treatment with mexiletine eliminated PACs and AT/AF in WT-HFD mice (Fig. 2A–C). These data provide the first evidence that dysregulation of voltage-gated sodium channels contributes to HFD-induced AF.

### SA mice are resistant to HFD-induced cardiac remodeling

**Fig. 3:**
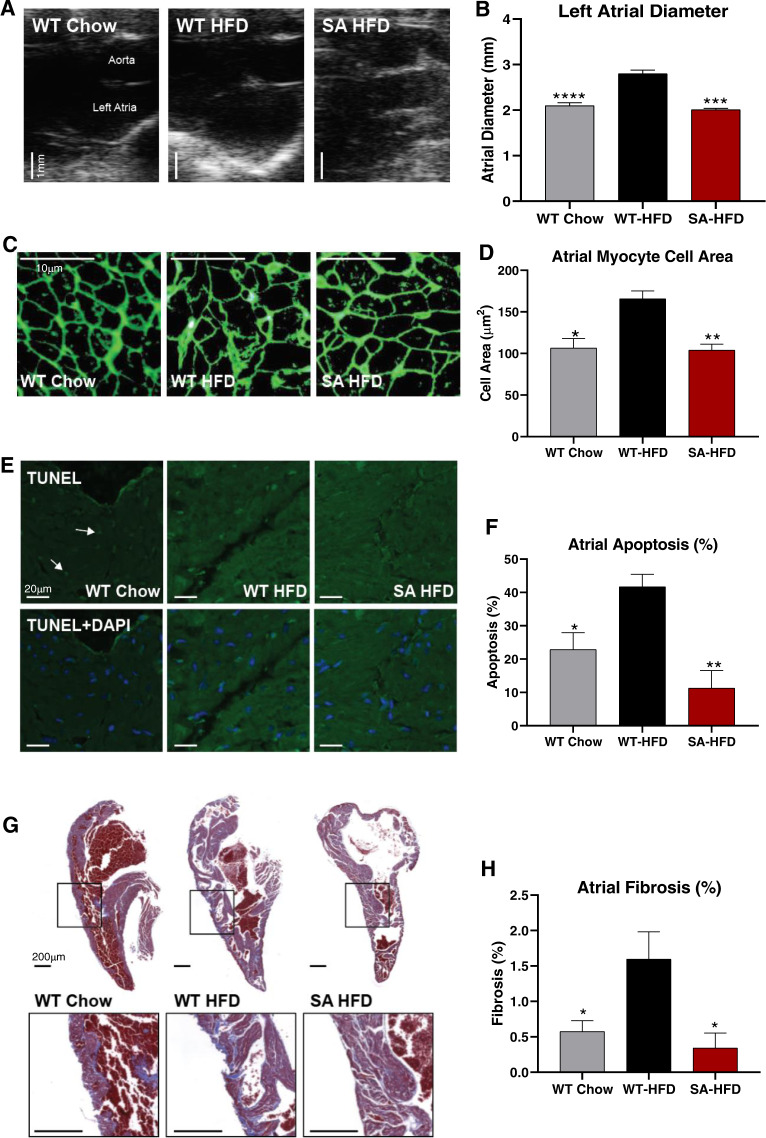
SA knock-in mouse model is resistant to high-fat diet-induced cardiac remodeling. **A** Representative echocardiograms (Scale bar 1 mm) and **B** quantification of left atrial diameters. Data are presented as means ± SEM (WT Chow *n* = 15, WT-HFD *n* = 15, SA-HFD *n* = 4; ***p < 0.001, ****p < 0.0001 vs WT-HFD). **C** Left atrial section cell membranes stained with Wheat germ agglutinin (Scale bar 10 μm) and **D** Cross-sectional areas measured from atrial cells to determine cell areas changes. Data are presented as means ± SEM (*n* = 3/group; *p < 0.05, **p < 0.01 vs WT-HFD). **E** TUNEL staining of left atrial sections to determine apoptosis (hot spots = nuclei with DNA fragmentation due to apoptosis; Arrows = toward hot spots; DAPI stain = location of all nuclei) (Scale bar 20 μm) and **F** Percentage of cells undergoing apoptosis. Data are presented as means ± SEM (WT Chow *n* = 4, WT-HFD *n* = 4; SA-HFD *n* = 3; *p < 0.05, **p < 0.01 vs WT-HFD). **G** Masson’s Trichrome staining of Left atrial sections to indicate fibrosis levels relative to normal cardiac tissue (Scale bar 200 μm) and **H** percentage of fibrotic tissue. Data are presented as means ± SEM (WT Chow *n* = 6, WT-HFD *n* = 6; SA-HFD *n* = 4; *p < 0.05 vs WT-HFD).

Previous studies have shown that obesity or a high-fat diet contribute to atrial remodeling and susceptibility to arrhythmias [17, 19, 21]. To investigate whether phospho-ablation of Na_v_1.5 protects against HFD-induced atrial remodeling, atrial size and structure were analyzed by echocardiography and immunohistochemistry. Echocardiography data revealed that HFD increased atrial chamber size in WT-HFD, but not SA-HFD mice. In fact, SA-HFD mice and WT Chow mice had similar atrial chamber size (Fig. 3A, B). HFD also increased atrial myocyte cross-sectional area in WT, but not SA mice (Fig. 3C, D). These data indicate that HFD induces pathological atrial hypertrophy in WT mice, while SA mice are resistant to this adverse remodeling.

In addition to pathological remodeling, AF is associated with increased apoptosis and tissue fibrosis. To determine if a high-fat diet affected atrial myocyte apoptosis and fibrosis, TUNEL staining was performed in WT Chow, WT-HFD, and SA-HFD mice. WT-HFD mice had increased apoptosis (Fig. 3E, F) and fibrosis (Fig. 3G, H) compared to WT Chow mice. Moreover, SA mice were resistant to the HFD-induced increase in apoptosis and fibrosis (Fig. 3E–H). These data indicate that phosphorylation of Na_v_1.5 is an important contributor to the development of HFD-induced atrial apoptosis and fibrosis. Together, these data demonstrate that phospho-ablation of the Na_v_1.5 channel negates HFD-induced atrial remodeling and arrhythmias.

### SA mice have improved metabolic capacity

SA mice are protected from HFD-induced arrhythmias and atrial remodeling (Figs. 1 and 3). It is important to note that these findings are confounded by significant differences in body weight between the WT-HFD and SA-HFD mice (Fig. 1), which are also present in WT and SA chow-fed mice (Supplementary Fig. 2A). This difference is likely due to altered food consumption as SA mice consumed significantly less food compared to WT mice on a chow diet (Supplementary Fig. 2B). Glucose tolerance was improved in SA chow-fed mice compared to WT chow-fed mice when injected with glucose based on either body weight (Supplementary Fig. 2C, D) or lean mass (Supplementary Fig. 2E, F). There was no difference in insulin tolerance or pyruvate tolerance between SA and WT mice on a chow diet (Supplementary Fig. 2G–J). These data indicate that attenuation of *I*_Na,L_ can affect metabolic capacity even on a chow diet.

**Fig. 4:**
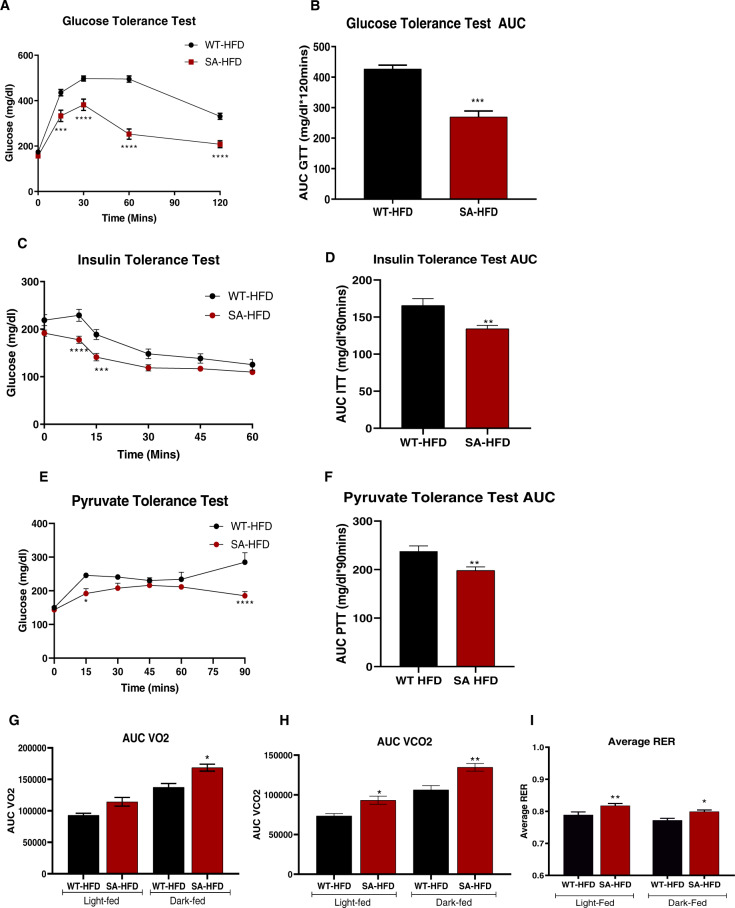
SA knock-in mouse model has improved metabolic capacity under conditions of diet-induced obesity. **A** Glucose tolerance test excursion curve and **B** glucose tolerance test area under curve after 6 weeks of HFD. Data are presented as means ± SEM (*n* = 5/group; **p < 0.01, ***p < 0.001, ****p < 0.0001 vs WT-HFD). **C** Insulin tolerance test excursion curve and **D** insulin tolerance test area under curve after 6 weeks of HFD. Data are presented as means ± SEM (*n* = 5/group; **p < 0.01, ***p < 0.001, ****p < 0.0001 vs WT-HFD). **E** Pyruvate tolerance test excursion curve and **F** pyruvate tolerance test area under curve after 6 weeks of HFD. Data are presented as means ± SEM (WT-HFD *n* = 5, SA-HFD *n* = 9; *p < 0.05, **p < 0.01, ****p < 0.0001 vs WT-HFD). **G** Volume of O_2_ consumption **H** volume of CO_2_ production, and **I** respiratory exchange ratio of mice was measured and calculated using CLAMS monitoring system as described previously after 12 weeks of HFD [27]. Data are presented as means ± SEM (WT-HFD *n* = 5, SA-HFD *n* = 4; *p < 0.05, **p < 0.01 vs WT-HFD).

SA-HFD mice were protected from diet-induced obesity and AF. Since metabolic dysfunction is a co-morbidity for obesity and AF, we investigated the effects of a high-fat diet on the metabolic health of SA mice. After 6 weeks of a high-fat diet, SA mice (SA-HFD) had a preserved glucose tolerance when injected with glucose based on body weight (Fig. 4A, B) or lean mass (Supplementary Fig. 3A, B), preserved insulin tolerance (Fig. 4C, D), and pyruvate tolerance (Fig. 4E, F) compared to high-fat fed wild-type mice (WT-HFD). SA-HFD mice had reduced fat mass and lean mass after 6 weeks of HFD compared to WT-HFD mice (Supplementary Fig. 3C–F). We measured metabolic capacity after 12 weeks of a high-fat diet via indirect calorimetry and determined that SA mice had increased VO_2_, VCO_2_, and respiratory exchange ratio (RER) (Fig. 4G–I), indicating that these mice predominantly used carbohydrates as a fuel source. These data indicate that preventing CaMKII-dependent phosphorylation of Na_v_1.5 leads to improved metabolic health in addition to having a protective effect on atrial remodeling and reduced susceptibility to arrhythmia.

Since the SA mice are a whole-body knock-in mouse, we investigated gene expression changes in several tissues to determine a potential role in mediating whole-body metabolic health. Expression of genes involved in mitochondrial metabolism, fatty acid oxidation, and glucose metabolism were measured in the liver, tibialis anterior (TA) skeletal muscle, interscapular brown adipose tissue (iBAT), inguinal subcutaneous white adipose tissue (ingWAT), and the small intestine. These tissues were selected for their documented roles in metabolism, and specifically glucose metabolism [25, 29, 30]. The small intestine was investigated because it expresses Na_v_1.5 in rodents and humans [31–33]. Select genes related to mitochondrial metabolism and fatty acid oxidation were increased in the liver, TA, iBAT, ingWAT, and small intestine of SA-HFD mice compared to WT-HFD mice (Supplementary Fig. 4A–O). Interestingly, there were no changes in expression of genes involved in glucose metabolism in any of the tissues measured (Supplementary Fig. 4A–O). These data indicate that some genes involved in mitochondrial and fatty acid metabolism are altered in multiple peripheral tissues in the SA mice and may contribute to whole-body changes in metabolism.

### Differences in body weight account for improved AT/AF susceptibility, but not metabolism in SA-HFD mice

**Fig. 5:**
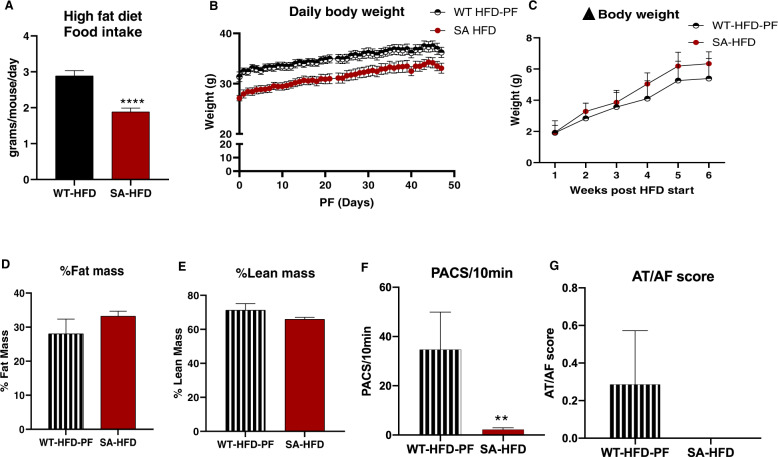
Attenuating body weight gain on HFD partially mitigates susceptibility to AF in wild-type mice compared to SA knock-in mice. **A** Average daily food intake of WT-HFD and SA-HFD mice on a high-fat diet. Data are presented as means ± SEM (WT-HFD *n* = 15, SA-HFD *n* = 5; ****p < 0.0001 vs WT-HFD). Wild-type mice were pair-fed (WT-HFD-PF) the same amount of HFD as SA-HFD mice for 6 weeks and **B** body weight **C** changes in body weight measured. **D** changes in percent fat and **E** lean mass measured using EchoMRI. Data are presented as means ± SEM (WT-HFD-PF *n* = 9, SA-HFD *n* = 10). **F** Density of pre-atrial contractions and **G** severity of AT/AF based on a score of 0 (none) to 4 (severe) measured following injection of epinephrine (1.5 mg/kg) and caffeine (120 mg/kg). Data are presented as means ± SEM (WT-HFD-PF *n* = 7, SA-HFD *n* = 14; **p < 0.01 vs WT-HFD-PF).

SA-HFD mice have reduced susceptibility to arrhythmias and improved metabolic health compared to WT-HFD mice. It is important to note that these findings are confounded by significant differences in body weight (Fig. 1A, B). Similar to a chow-fed mice, SA mice had reduced food intake compared to WT mice on HFD (Supplementary Fig. 2B, Fig. 5A). To determine if the differences in food intake were the primary driver for the difference in body weight, resistance to arrhythmia development, and improved metabolic health in SA-HFD mice, we investigated a separate cohort of mice where food intake in WT-HFD mice was paired to that of SA-HFD mice (WT-HFD-PF) (Supplementary Fig. 5A). Pair-feeding blunted the HFD-induced weight gain observed in *ad libitum* fed WT mice (Fig. 5B, C; Supplementary Fig. 5B, C). Pair-feeding also abolished the differences in fat mass (Supplementary Fig. 5D), percent fat mass (Fig. 5D), and percent lean mass (Fig. 5E), but not total lean mass (Supplementary Fig. 5E), between WT-HFD and SA-HFD mice. In parallel, pair-feeding reduced the difference in fibrosis between the both groups compared to WT-HFD, although a significant increase in fibrosis was still apparent in WT-HFD-PF compared to SA-HFD (Supplementary Fig. 5F, G).

To determine if susceptibility to AT/AF was primarily in response to changes in body weight, WT-HFD-PF mice were compared to *ad libitum* fed WT-HFD mice, WT Chow mice (Fig. 1C, D), and SA-HFD mice. While pair-feeding did not eliminate differences in PACs between WT-HFD-PF and SA-HFD mice (Fig. 5F), pair-feeding reduced incidence of AT/AF in WT-HFD-PF mice (Fig. 5G). WT-HFD-PF mice had a lower AT/AF score compared to WT-HFD mice (Figs. 1D and 5G), with no significant differences in AT/AF score between WT-HFD-PF, WT Chow, or SA-HFD mice (Figs. 1D and 5G). These data indicate that the SA allele reduces severity of atrial arrhythmia events dependent on food consumption and weight gain.

**Fig. 6:**
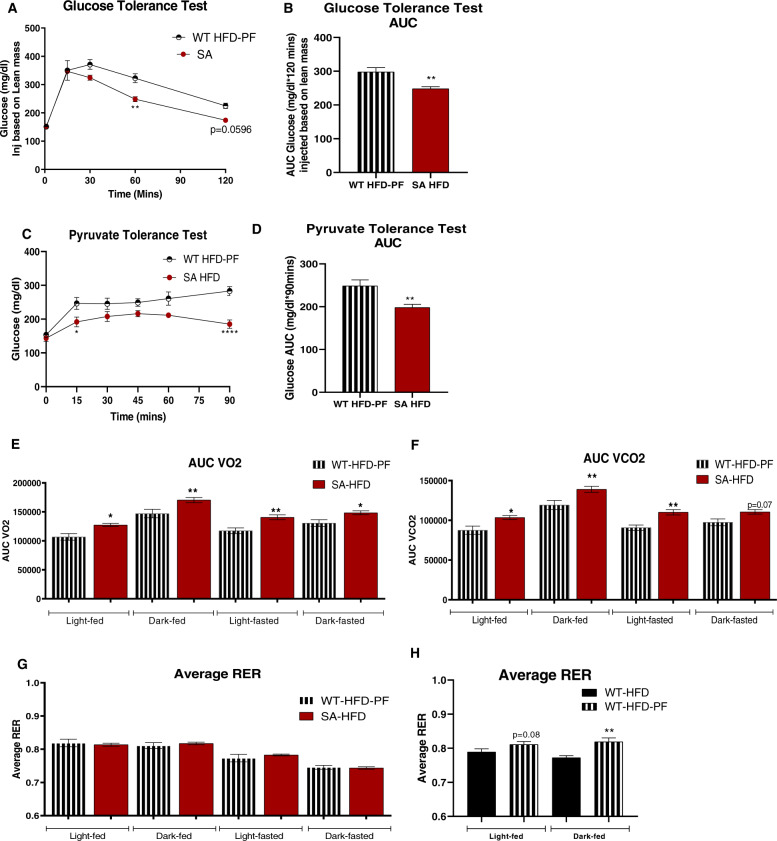
Attenuating body weight gain on HFD does not affect metabolic capacity of wild-type mice compared to SA knock-in mice. **A** Glucose tolerance test excursion curve and **B** area under curve for glucose tolerance after 6 weeks of pair-feeding. **C** Pyruvate tolerance excursion curve and **D** area under curve for pyruvate tolerance after 6 weeks of pair-feeding. Data are presented as means ± SEM (WT-HFD-PF *n* = 9, SA-HFD *n* = 10; *p < 0.05, **p < 0.01, ****p < 0.0001 vs WT-HFD-PF). **E** Volume of O_2_ consumption; **F** volume of CO_2_ production; **G**, **H** respiratory exchange ratio of mice measured using CLAMS Monitoring system after 6 weeks of pair feeding [27]. Data are presented as means ± SEM WT-HFD *n* = 5, WT-HFD-PF *n* = 9, SA-HFD *n* = 10; *p < 0.05, **p < 0.01 vs WT-HFD-PF (**E**–**G**) and vs WT-HFD (**H**).

To determine if the observed metabolic improvements were dependent on body weight in SA-HFD mice, we investigated the metabolic health of WT-HFD-PF mice compared to SA-HFD mice. To account for the slight differences in body weight, mice were injected with glucose based on lean mass and glucose tolerance was measured. Even when injected based on lean mass, glucose tolerance was improved in SA-HFD compared to WT-HFD-PF mice (Fig. 6A, B). Insulin tolerance was not different between groups (Supplementary Fig. 5H, I), but pyruvate tolerance was improved in SA-HFD mice, indicating that SA-HFD mice had improved gluconeogenic activity compared to WT-HFD-PF mice (Fig. 6C, D). Together these data indicate that the effects of improved glucose and pyruvate tolerance were not dependent on body weight. Indirect calorimetry measurements revealed that SA-HFD mice had increased VO_2_ and VCO_2_ compared to WT-HFD-PF mice, but with no difference in RER (Fig. 6E–G). Interestingly, WT-HFD-PF mice had increased RER compared to *ad libitum* WT-HFD mice (Fig. 6H), indicating a shift to increased carbohydrate utilization in these mice, similar to SA-HFD mice. Taken together, these data indicate that SA-HFD mice have systemic improvements in metabolic health compared to WT-HFD-PF mice that are independent of body weight.

## Discussion

In this study, we investigated the link between CaMKII-dependent regulation of Na_v_1.5, pathogenic late current (*I*_Na,L_), diet-induced obesity, and AF, taking advantage of our phospho-ablated SA knock-in mouse model lacking the CaMKII phosphorylation site on Na_v_1.5, which attenuates *I*_Na,L_. Diet-induced obesity resulted in increased arrhythmias and obesity-induced atrial remodeling (fibrosis, pathological hypertrophy, apoptosis) in WT mice, but phospho-ablation of Na_v_1.5 in the SA knock-in mice conferred resistance to obesity-induced arrhythmias and atrial remodeling. Moreover, phospho-ablation of Na_v_1.5 resulted in improved metabolic capacity in mice, independent of body weight. This highlights a novel role for the late Na^+^ current to modulate whole-body metabolism.

Dysregulation of Na_v_1.5 is frequently reported in animal models of AF and human patients [8, 34–37]. Our group and others have demonstrated that dysregulation of Na_v_1.5 mediates atrial arrhythmogenesis downstream of CaMKII activation [8, 10, 12]. Of specific interest, previous work has shown that in addition to other targets involved in AF pathogenesis, CaMKII phosphorylates Na_v_1.5 at Ser571 in both atrial and ventricular myocytes, directly augmenting *I*_Na,L_ and disrupting intracellular homeostasis of both Na^+^ and Ca^2+^ [8, 10, 38]. Dysregulation of Na_v_1.5 Ser571 has been observed in multiple cardiac disease states, including heart failure, ischemia/reperfusion, and AF [38–40]. In addition, drugs like ranolazine that target *I*_Na,L_ have been successfully employed to treat AF [34, 41, 42]. However, the role of *I*_Na,L_ in stress-induced AF, such as that resulting from aging, heart failure, and obesity, remains unclear. An outstanding question to be answered going forward is how does a high-fat diet promotes CaMKII/Na_v_1.5 dysregulation and atrial structural/electrical remodeling to set the stage for increased susceptibility to atrial arrhythmias? Previous work has shown increased CaMKII activity with a high-fat diet downstream of increased oxidative stress [43]. At the same time, accumulation of epicardial adipose tissue has been linked to atrial remodeling through paracrine signaling [44, 45]. Thus it is likely that changes in the volume/composition of fat depots adjacent to the atria may drive the remodeling process and arrhythmia, although further investigation is needed.

Here, we determined that HFD increased atrial arrhythmia burden in WT mice, but preventing augmentation of *I*_Na,L_ in SA reduced susceptibility to the development of arrhythmias. Surprisingly, SA mice also had an improved metabolic profile compared to WT-HFD mice, independent of their body weight. These improvements were similar to previous studies investigating the role of the *I*_Na,L_ inhibitor ranolazine. The HARMONY and RAFFAELLO trials have shown that ranolazine decreases AF burden and recurrence in AF patients [46, 47]. In addition, pre-clinical and clinical studies have reported that ranolazine induces weight loss and improves glycemia in patients with type 2 diabetes and coronary heart disease [48–50]. Although the mechanism remains unclear, it has been proposed that ranolazine may alter metabolism by reducing glucagon release from the pancreas, or by modulating fatty acid uptake, oxidation, and gluconeogenesis in the liver [51–53]. Based on our findings, it is possible that metabolic benefits of Na_v_1.5 phospho-ablation and ranolazine are mediated through similar mechanisms.

While Na_v_1.5 is predominantly expressed in the heart, Na_v_1.5 is also found in other tissues [54, 55]. The impact of Na_v_1.5 on glucose metabolism in tissues other than the heart has not been studied. Since the SA mouse model is a whole-body knock-in model, effects on peripherally expressed Na_v_1.5 could mediate the observed metabolic benefits. Analysis of genes in the liver, TA, iBAT, ingWAT, and small intestine indicates some changes to mitochondrial and glucose metabolism in SA-HFD mice that warrant further studies to better determine their role in improving metabolic capacity.

It is important to note that SA mice had lower food intake on HFD compared to WT mice which reduced the weight gain in these animals. Attenuating body weight gain by pair-feeding WT and SA mice eliminated differences in HFD-induced AT/AF but not PACs in WT mice compared to SA mice. This indicates that reduced body weight gain likely reduces the severity of the AF disease state, but not arrhythmogenic triggers factors like PACs. In contrast, improved glucose metabolism was still observed in SA-HFD mice compared to WT-HFD-PF, suggesting a direct effect of Na_v_1.5 on metabolism. Future studies should investigate the impact of therapeutic treatments such as ranolazine on food intake in patients, as well as mechanisms behind decreased food intake and its role in metabolic effects of Na_v_1.5. Moreover, it will be of value to highlight the relative importance of modulating cardiac-specific changes in Na_v_1.5 activity, relative to extra-cardiac Na_v_1.5. Interestingly, the use of mexiletine successfully reduced AF burden in WT-HFD mice with just 15 min of pre-treatment, suggesting a critical importance of direct electrophysiologic action of Ser571 in acutely contributing to AF burden. It will be interesting to see how this differs from the chronic contributions of Ser571 to the structural changes of atrial hypertrophy and fibrosis and how such changes incorporate extra-cardiac Na_v_1.5. It is also worth noting that cardiac-specific transgenic models have altered systemic energy homeostasis, suggesting that modulation of cardiac Nav1.5 alone may be sufficient to induce non-cardiac changes in metabolic regulation and energy storage [56].

Overall, these data demonstrate a previously understudied interaction between the CaMKII/Na_v_1.5 pathway, AF, and obesity. Phospho-ablation of the Na_v_1.5 site, Ser571, that regulates *I*_Na,L_ reduces AF susceptibility by attenuating weight gain and improves whole-body glucose metabolism independent of body weight. Together, these data establish Na_v_1.5 as an important driver for whole-body metabolic health and reiterate its importance in arrhythmic susceptibility under conditions of diet-induced obesity. These data also highlight the need to better elucidate the mechanistic basis for observed changes to metabolism and arrhythmia susceptibility with future studies. This study provides greater understanding of the interplay between co-morbidities like cardiovascular diseases, obesity, type 2 diabetes and their underlying effectors. Analysis of the mechanistic aspects of these diseases, especially the role of the late Na^+^ current in this context, can further the understanding and treatment of these co-morbidities in humans.

## Supplementary information

Supplemental Figure Legends, Supplemental Tables

Supplemental Figs. 1-5
