# Supplementary material for: Phospho-ablation of cardiac sodium channel Nav1.5 mitigates susceptibility to atrial fibrillation and improves glucose homeostasis under conditions of diet-induced obesity
Source: Int J Obes (Lond). 2021 Jan 26;45(4):795–807. doi: 10.1038/s41366-021-00742-4 (PMC8005377; doi:10.1038/s41366-021-00742-4)

Supplementary Figure 1. Six weeks of high-fat diet upregulates CaMKII and Na_v1.5 phosphorylation in WT-HFD mice. Representative immunoblots and densitometry showing (**A**) phosphorylated Na_v1.5 at S571 (pNav1.5) relative to total Na_v1.5 and (**B**) phosphorylated CaMKII at T286/287 (pCaMKII) relative to total CaMKII after 6 weeks of HFD. Data represented as means ± SEM (WT-chow n=4, WT-HFD n=9, SA-HFD n=4; *p<0.05 vs WT-HFD)

Supplementary Figure 2. SA mice have reduced weight, food intake and improved glucose tolerance compared to WT mice on a chow diet. (**A**) Body weight of WT and SA mice on a chow diet. Data are presented as means ± SEM (WT Chow n=22, SA-chow n=11; ****p<0.0001 vs WT chow). (**B**) Average daily food intake of WT and SA mice on a chow diet. Data are presented as means ± SEM (WT chow n=3, SA chow n=3; **p<0.01 vs WT chow) (**C**) Glucose tolerance test excursion curve and (**D**) glucose tolerance test area under curve. Data are presented as means ± SEM (n=5/group; *p<0.05 vs WT chow). (**E**) Glucose tolerance test excursion curve and (**F**) glucose tolerance test area under curve. Data are presented as means ± SEM (n=5/group; *p<0.05, **p<0.01 vs WT chow) (**G**) Insulin tolerance test excursion curve and (**H**) insulin tolerance test area under curve. Data are presented as means ± SEM (n=5/group; **p<0.01 vs WT chow). (**I**) Pyruvate tolerance test excursion curve and (**J**) pyruvate tolerance test area under curve after 6 weeks of HFD. Data are presented as means ± SEM (WT-HFD n=5, SA-HFD n=9; *p<0.05, **p<0.01, ****p<0.0001 vs WT-HFD)

Supplementary Figure 3. Six weeks of high-fat diet causes changes in glucose tolerance and body composition of both WT-HFD and SA-HFD mice. (**A**) Glucose

tolerance test excursion curve. Data are presented as means ± SEM (WT chow n=10, WT-HFD n=25, SA-HFD n=12; *p<0.05, **p<0.01, ***p<0.001, ****p<0.0001 for WT chow vs. WT-HFD; $^{\$}$p<0.05, $^{\$\$}$p<0.01, $^{\$\$\$}$p<0.001, $^{\$\$\$\$}$p<0.0001). (**B**) Glucose tolerance test area under curve. Data are presented as means ± SEM (WT chow n=10, WT-HFD n=25, SA-HFD n=12; ***p<0.001 vs. WT-HFD. Changes in (**C**) total fat mass, (**D**) percent fat mass, (**E**) total lean mass and (**F)** percent lean mass after 6 weeks of chow diet or HFD were measured using EchoMRI**.** Data are presented as means ± SEM (WT-Chow n=10, WT-HFD n=20, SA-HFD n=12; **p<0.01, ****p<0.0001 vs WT-HFD).

Supplementary Figure 4. Expression of genes related to mitochondrial metabolism, fatty acid oxidation and glucose metabolism in various tissues in SA-HFD mice. Gene expression analysis for **Liver** (**A**) Mitochondrial metabolism (**B**) Fatty acid oxidation (**C**) Glucose metabolism, **TA** (**D**) Mitochondrial metabolism (**E**) Fatty acid oxidation (**F**) Glucose metabolism, **iBAT** (**G**) Mitochondrial metabolism (**H**) Fatty acid oxidation (**I**) Glucose metabolism, **ingWAT** (**J**) Mitochondrial metabolism (**K**) Fatty acid oxidation (**L**) Glucose metabolism and **small intestine** (**M**) Mitochondrial metabolism (**N**) Fatty acid oxidation (**O**) Glucose metabolism. Data are presented as means ± SEM (n=5/group; *p<0.05, **p<0.01, ***p<0.001, ****p<0.0001 vs WT-HFD)

Supplementary Figure 5. Pair-feeding of WT mice (WT-HFD-PF) to SA-HFD mice over 6 weeks of HFD (A) Schematic description of pair-feeding WT-HFD-PF mice the amount of food consumed by SA-HFD mice over 6 weeks of diet. (**B**) Daily food intake and (**C**) average food intake for all WT-HFD-PF mice was measured. (**D**) Changes in total fat mass and (**E**) total lean mass were measured using EchoMRI. Data are represented as Mean ± SEM (WT-HFD-PF n=9, SA-HFD n=10; **p<0.01 vs WT-HFD-

PF) (**F**) Masson's Trichrome staining of left atrial sections to indicate fibrosis (blue) levels relative to normal cardiac tissue (red) (Scale bar 200 µm) and (**G**) Percentage of fibrotic tissue normalized to WT. Data are presented as means ± SEM (SA-HFD n=3; WT-HFD-PF n=5; *p<0.05). (**H**) Insulin tolerance and (**I**) area under curve for insulin tolerance was measured by injecting insulin 1 unit per kg body weight i.p. Data are represented as Mean ± SEM (WT-HFD-PF n=9, SA-HFD n=10; **p<0.01 vs WT-HFD-PF).

Supplementary Table 1: qPCR forward and reverse primer sequences for the genes measured, related to Supplemental Figure 4.

Gene	Forward Primer Sequence	Reverse Primer Sequence
Acsl1	ACACTTCCTTGAAGCGATGG	GGCTCGACTGTATCTTGTGG
Acsl3	GGCCAACGTGGAAAAGAAAG	GTCTTGGAATCCTTCTCGCC
Acsl4	GCACCTTCGACTCAGATCAC	CCAGGTTTGTCTGAAGTGGG
Acsl5	CGCCCCCATCTCCACTCCAG	GCTTCAAACACCCAACATCCCATTGC
Aldoa	GCGACCACCATGTCTATCTG	GAAAGTGACCCCAGTGACAG
Aldoa	GCGACCACCATGTCTATCTG	GAAAGTGACCCCAGTGACAG
Cd36	TGGAGCTGTTATTGGTGCAG	TGGGTTTTGCACATCAAAGA
Cd36	TGGAGCTGTTATTGGTGCAG	TGGGTTTTGCACATCAAAGA
Cidea	GGTGGACACAGAGGAGTTCTTTC	CGAAGGTGACTCTGGCTATTCC
Cidea	GGTGGACACAGAGGAGTTCTTTC	CGAAGGTGACTCTGGCTATTCC

Cpt1	AAAGATCAATCGGACCCTAGACA	CAG CGA GTA GCG CAT AGT CA
Cpt1a	AAAGATCAATCGGACCCTAGACA	CAG CGA GTA GCG CAT AGT CA
Cs	GACTACATCTGGAACACACTCAATTCA	CGAGGGTCAGTCTTCCTCAGTAC
Cs	GACTACATCTGGAACACACTCAATTCA	CGAGGGTCAGTCTTCCTCAGTAC
Elovl3	GTGCTTTGCCATCTACACGGATG	ATGAGTGGACGCTTACGCAGGA
Elovl3	GTGCTTTGCCATCTACACGGATG	ATGAGTGGACGCTTACGCAGGA
Eno1	CTTGCTTTGCAGCGATCCTA	GAAGAGACCTTTTGCGGTGT
Eno1	CTTGCTTTGCAGCGATCCTA	GAAGAGACCTTTTGCGGTGT
Eno3	CTGTGCCTGCCTTTAATGTG	CTTCCCATACTTGGCCTTGA
Eno3	CTGTGCCTGCCTTTAATGTG	CTTCCCATACTTGGCCTTGA
Fabp3	AGAGTTCGACGAGGTGACAGCA	TTGTCTCCTGCCCGTTCCACTT
Fabp3	AGAGTTCGACGAGGTGACAGCA	TTGTCTCCTGCCCGTTCCACTT
Fabp5	GACGACTGTGTTCTCTTGTAACC	TGTTATCGTGCTCTCCTTCCCG
Fabp5	GACGACTGTGTTCTCTTGTAACC	TGTTATCGTGCTCTCCTTCCCG
Fatp1	TGCCACAGATCGGCGAGTTCTA	AGTGGCTCCATCGTGTCCTCAT
Fatp4	GACTTCTCCAGCCGTTTCCACA	CAAAGGACAGGATGCGGCTATTG
Fbp1	TGCTGAAGTCGTCCTACGCTAC	TTCCGATGGACACAAGGCAGTC
Ffar4	GTGACTTTGAACTTCCTGGTGCC	CAGAGTATGCCAAGCTCAGCGT
Ffar4	GTGACTTTGAACTTCCTGGTGCC	CAGAGTATGCCAAGCTCAGCGT
G6Pc	AGGTCGTGGCTGGAGTCTTGTC	GTAGCAGGTAGAATCCAAGCGC

Gapdh	AACTTTGGCATTGTGGAAGG	ACACATTGGGGGTAGGAACA
Glut4	ATCATCCGGAACCTGGAGG	CGGTCAGGCGCTTTAGACTC
Glut4	ATCATCCGGAACCTGGAGG	CGGTCAGGCGCTTTAGACTC
Gpd1	CTCATCACGACCTGCTATGG	CTGCTCAATGGACTTTCCAG
Gpd2	GCGGACTCATCACAATAGCA	TGAAGGAACAGCCCAACAG
Gpi1	ATGGGCATATTCTGGTGGAC	CCCGATTCTCGGTGTAGTTG
Gpi1	ATGGGCATATTCTGGTGGAC	CCCGATTCTCGGTGTAGTTG
Gyk	CAAATGCAAGCAGGACGATG	GGCCCCAGCTTTCATTAGG
Hk2	AGAGAACAAGGGCGAGGAG	GGAAGCGGACATCACAATC
Hk2	AGAGAACAAGGGCGAGGAG	GGAAGCGGACATCACAATC
Idh3a	GCAGGACTGATTGGAGGTCTTG	GCCATGTCCTTGCCTGCAATGT
Lipe	TGGCACACCATTTTGACCTG	TTGCGGTTAGAAGCCACATAG
Lpl	GATGCCCTACAAAGTGTTCCA	AAATCTCGAAGGCCTGGTTG
Mdh2	TCACTCCTGCTGAAGAACAGCC	CCTTTGAGGCAATCTGGCAACTG
Nrf1	CAACAGGGAAGAAACGGAAA	GCACCACATTCTCCAAAGGT
Nrf1	CAACAGGGAAGAAACGGAAA	GCACCACATTCTCCAAAGGT
Nrf2	AGGTTGCCCACATTCCCAAACAAG	TTGCTCCATGTCCTGCTCTATGCT
Nrf2	AGGTTGCCCACATTCCCAAACAAG	TTGCTCCATGTCCTGCTCTATGCT
Ogdh	GGTGTCGTCAATCAGCCTGAGT	ATCCAGCCAGTGCTTGATGTGC
Pcx	GGATGACCTCACAGCCAAGCAT	GCAATCGAAGGCTGCGTACAGT

Pdha1	GTGAGAACAACCGCTATGGCATG	CGCAAACTTTGTTGCCTCTCGG
Pfkl	CCATCAGCAACAATGTGCCTGG	TGAGGCTGACTGCTTGATGCGA
Pfkl	CCATCAGCAACAATGTGCCTGG	TGAGGCTGACTGCTTGATGCGA
Pfkm	CTGGTGCTGAGGAATGAGAA	TTCCTGTCAAAGGGAGTTGG
Pfkm	CTGGTGCTGAGGAATGAGAA	TTCCTGTCAAAGGGAGTTGG
Pfkp	AAGCTATCGGTGTCCTGACC	TCCCACCCACTTGCAGAAT
Pfkp	AAGCTATCGGTGTCCTGACC	TCCCACCCACTTGCAGAAT
Pgam1	GACGATCTTATGATGTCCCACC	GTACCTGCGATCCTTGCTGA
Pgam1	GACGATCTTATGATGTCCCACC	GTACCTGCGATCCTTGCTGA
Pgam2	CTGCCTACCTGTGAAAGTCTC	GACATCCCTTCCAGATGTTT
Pgam2	CTGCCTACCTGTGAAAGTCTC	GACATCCCTTCCAGATGTTT
Pgc1a	GAATCAAGCCACTACAGACACCG	CATCCCTCTTGAGCCTTTCGTG
Pgc1a	GAATCAAGCCACTACAGACACCG	CATCCCTCTTGAGCCTTTCGTG
Pgc1b	TCCTGTAAAAGCCCGGAGTAT	GCTCTGGTAGGGGCAGTGA
Pgc1b	TCCTGTAAAAGCCCGGAGTAT	GCTCTGGTAGGGGCAGTGA
Pgk1	GAGCCTCACTGTCCAAACTA	CTTTAGCGCCTCCCAAGATA
Pgk1	GAGCCTCACTGTCCAAACTA	CTTTAGCGCCTCCCAAGATA
Pklr	CGAAAAGCCAGTGATGTGGTGG	GATGCCATCGCTCACTTCTAGG
Pkm	CTGTGGAGATGCTGAAGGAG	CAACAGGACGGTAGAGAATGG
Pkm	CTGTGGAGATGCTGAAGGAG	CAACAGGACGGTAGAGAATGG

Prdm16	ATCCACAGCACGGGTGAAGCCAT	ACATCTGCCCACAGTCCTTGCA
Prdm16	ATCCACAGCACGGGTGAAGCCAT	ACATCTGCCCACAGTCCTTGCA
Tfam	GTCCATAGGCACCGTATTGC	CCCATGCTGGAAAAACACTT
Tfam	GTCCATAGGCACCGTATTGC	CCCATGCTGGAAAAACACTT
Tpi1	TATGGAGGTTCTGTGACTGGA	CGGTGGGAGCAGTTACTAAA
Tpi1	TATGGAGGTTCTGTGACTGGA	CGGTGGGAGCAGTTACTAAA
Ucp1	AGGCTTCCAGTACCATTAGGT	CTGAGTGAGGCAAAGCTGATTT
Ucp1	AGGCTTCCAGTACCATTAGGT	CTGAGTGAGGCAAAGCTGATTT

Supplementary Figure 1

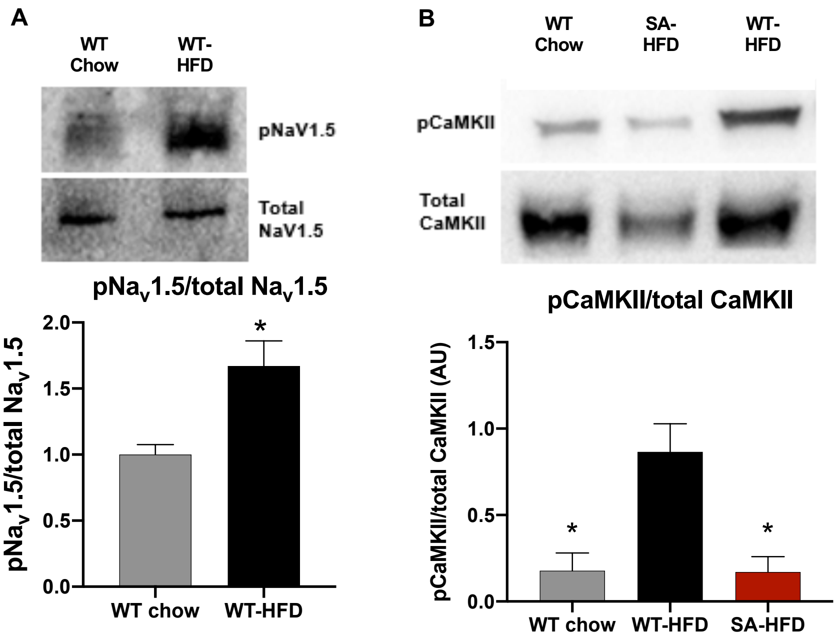

Supplementary Figure 2

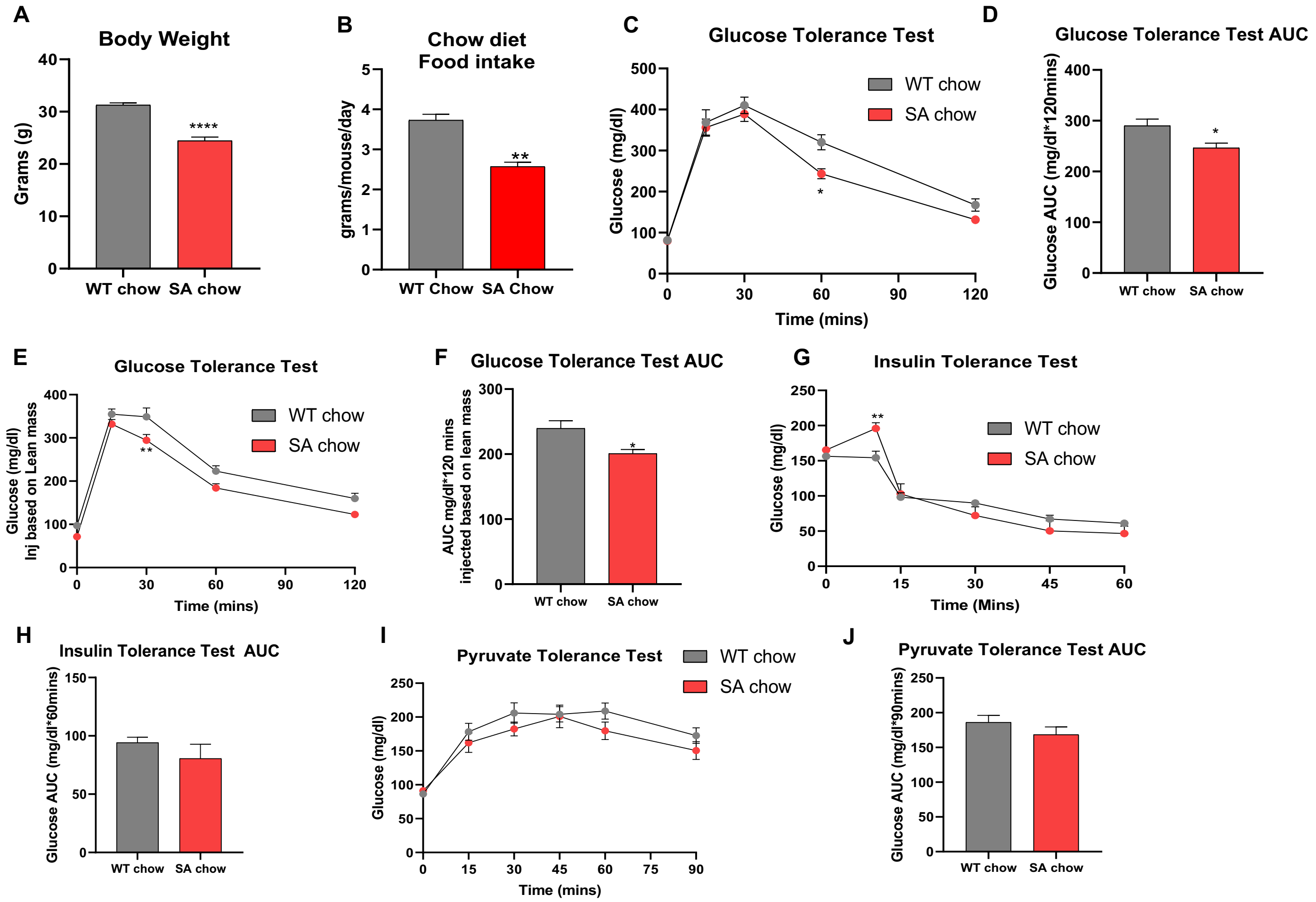

Supplementary Figure 3

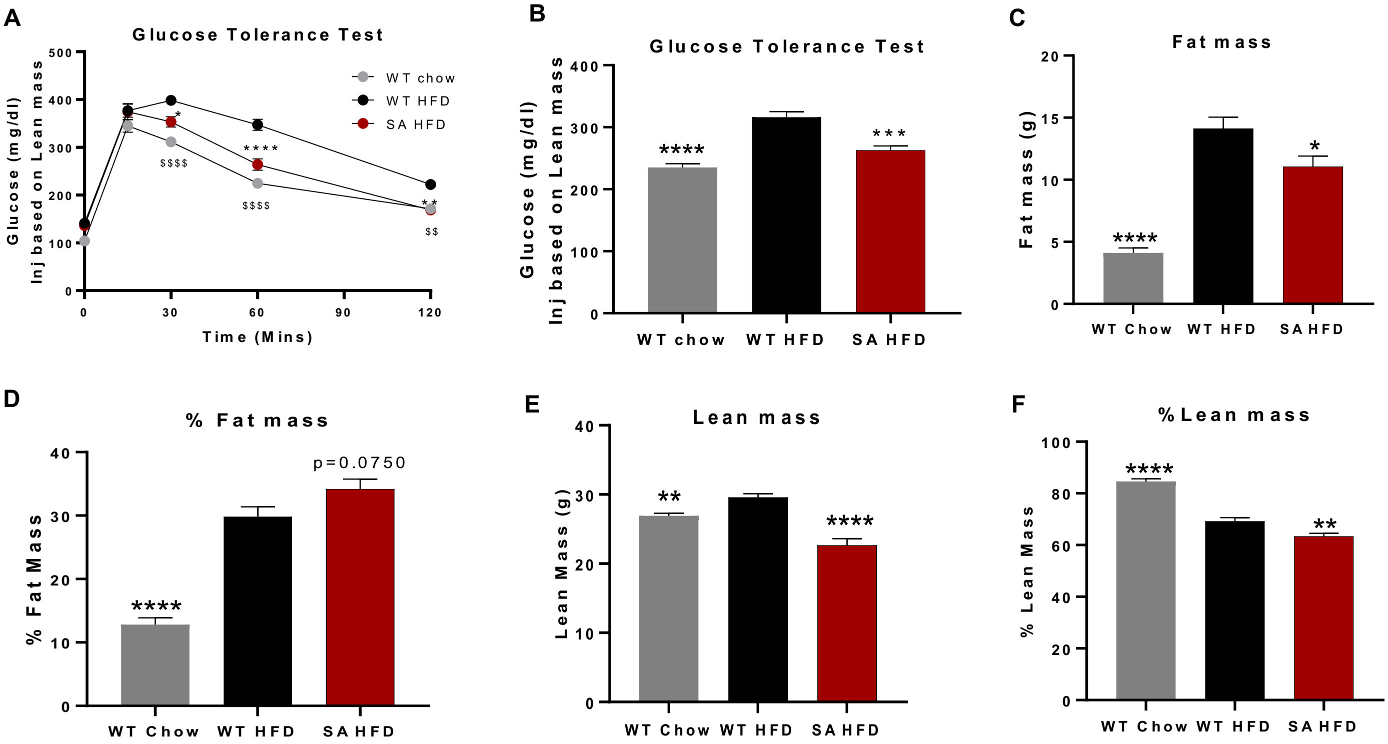

Supplementary Figure 4

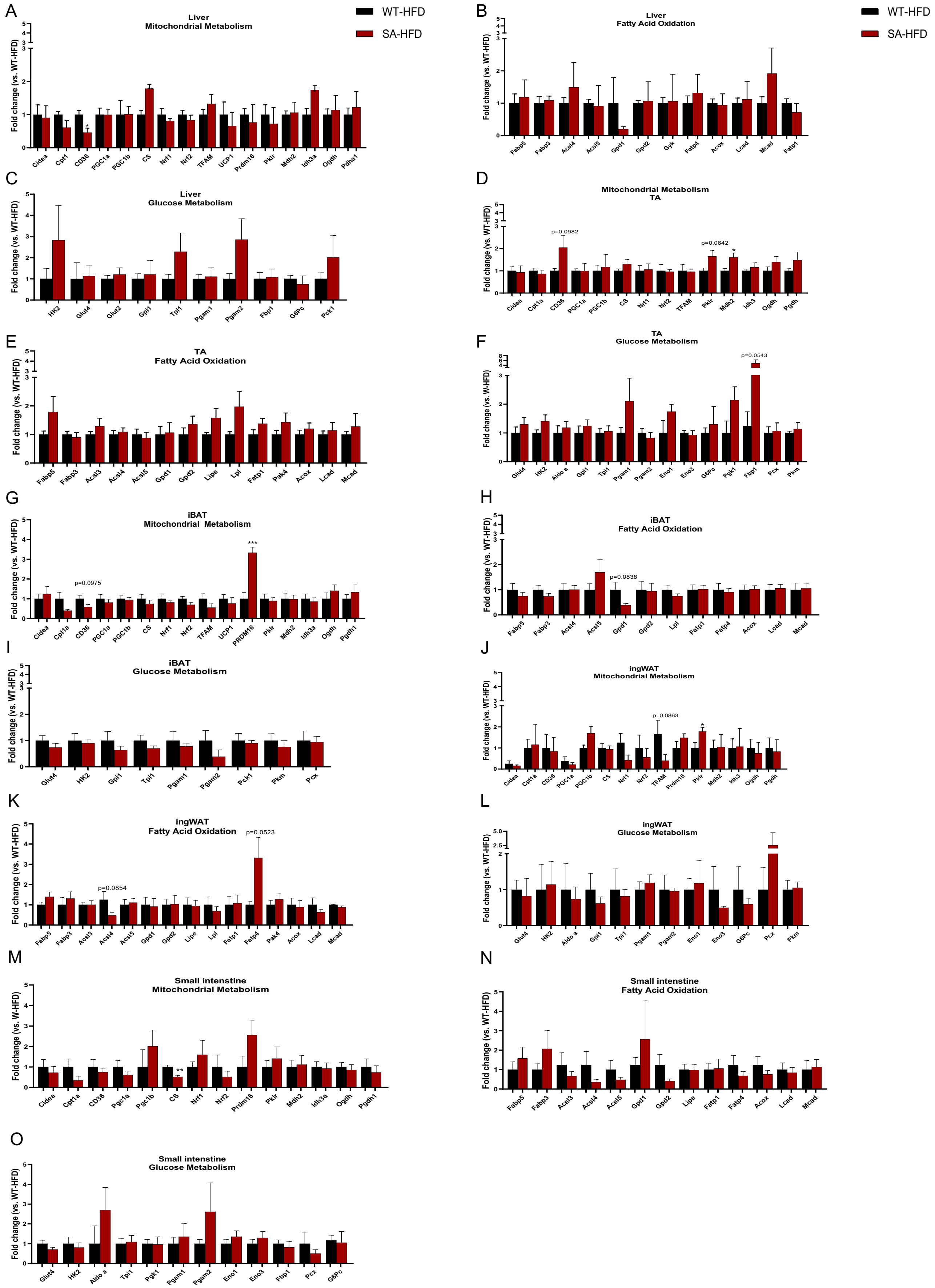

Supplementary Figure 5

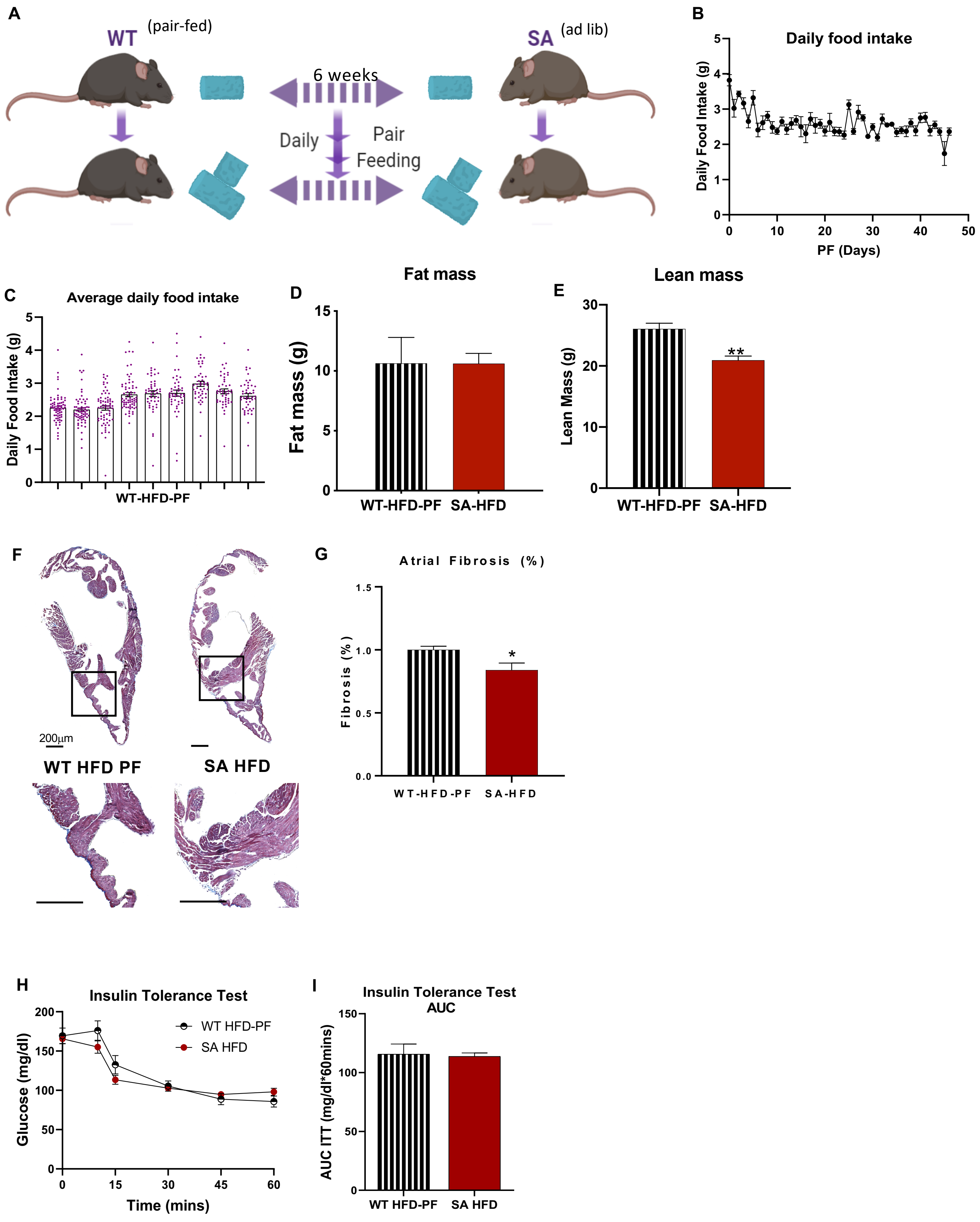

Supplement: Supplementary file 1 — Supplemental Figure Legends, Supplemental Tables [file 41366_2021_742_MOESM1_ESM.pdf]
