# Supplementary material for: Phospho-ablation of cardiac sodium channel Nav1.5 mitigates susceptibility to atrial fibrillation and improves glucose homeostasis under conditions of diet-induced obesity
Source: Int J Obes (Lond). 2021 Jan 26;45(4):795–807. doi: 10.1038/s41366-021-00742-4 (PMC8005377; doi:10.1038/s41366-021-00742-4)
Supplement: Supplementary file 2 — Supplemental Figs. 1-5 [file 41366_2021_742_MOESM2_ESM.pptx]

## Slide 1
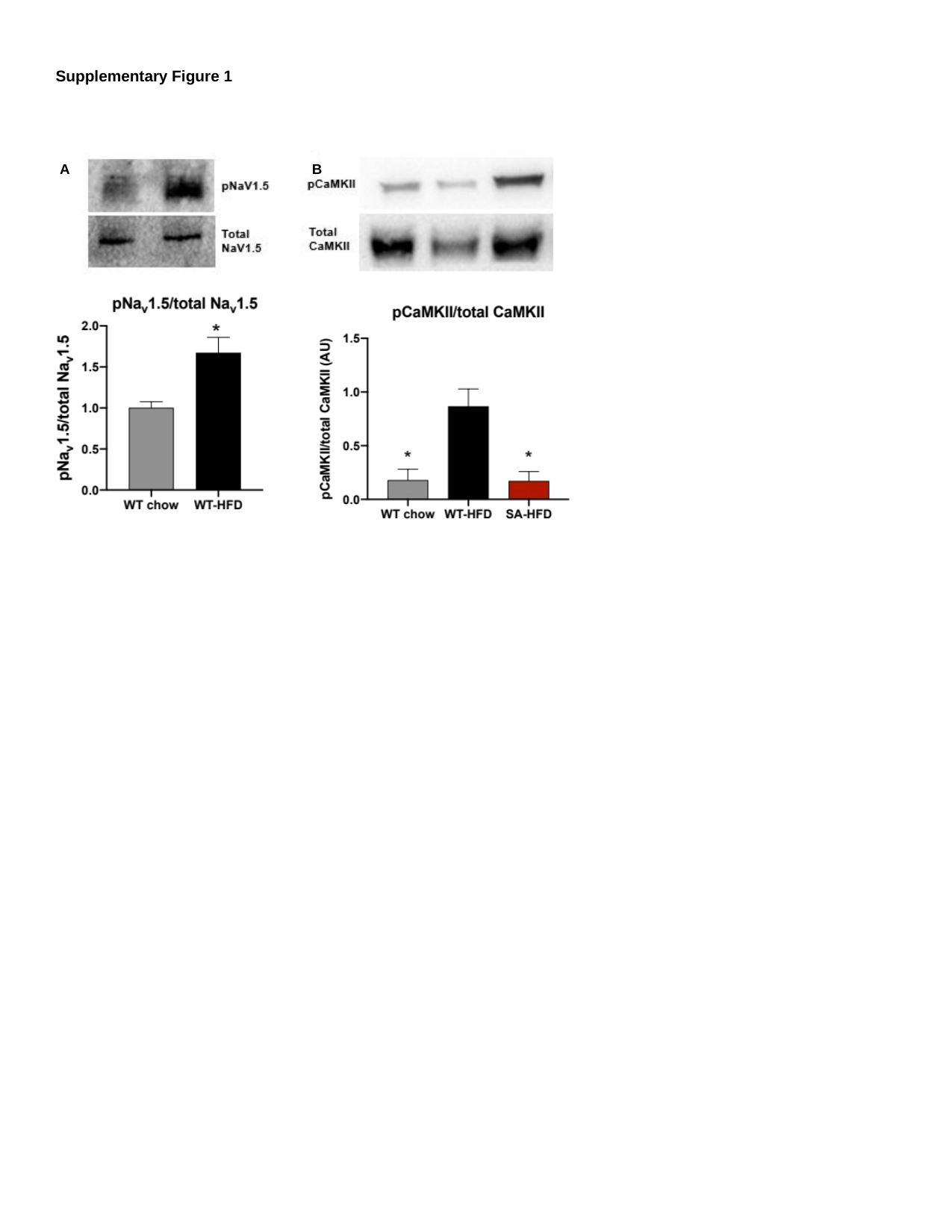

Supplementary Figure 1
B
A
B

## Slide 2
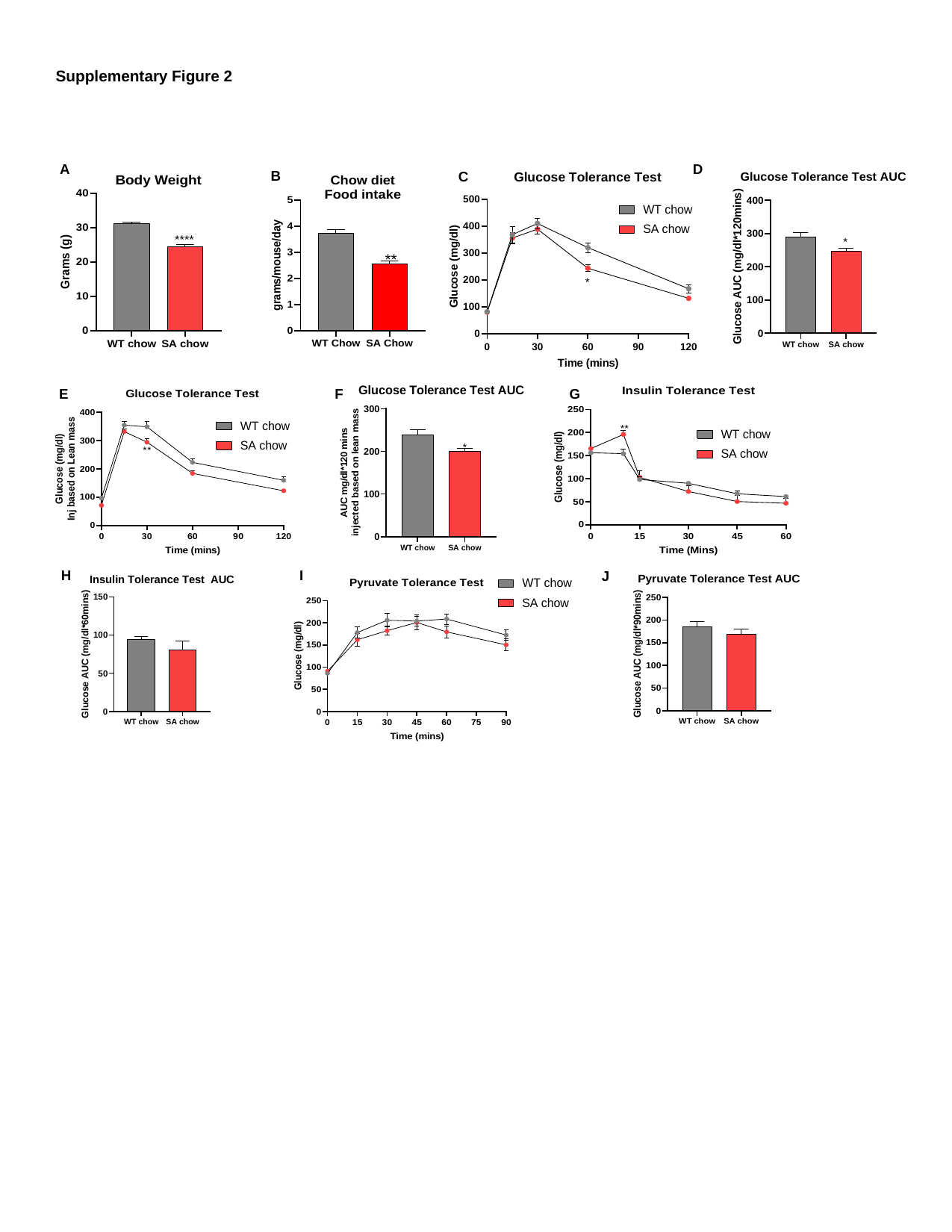

Supplementary Figure 2
A
D
B
C
E
F
G
H
I
J

## Slide 3
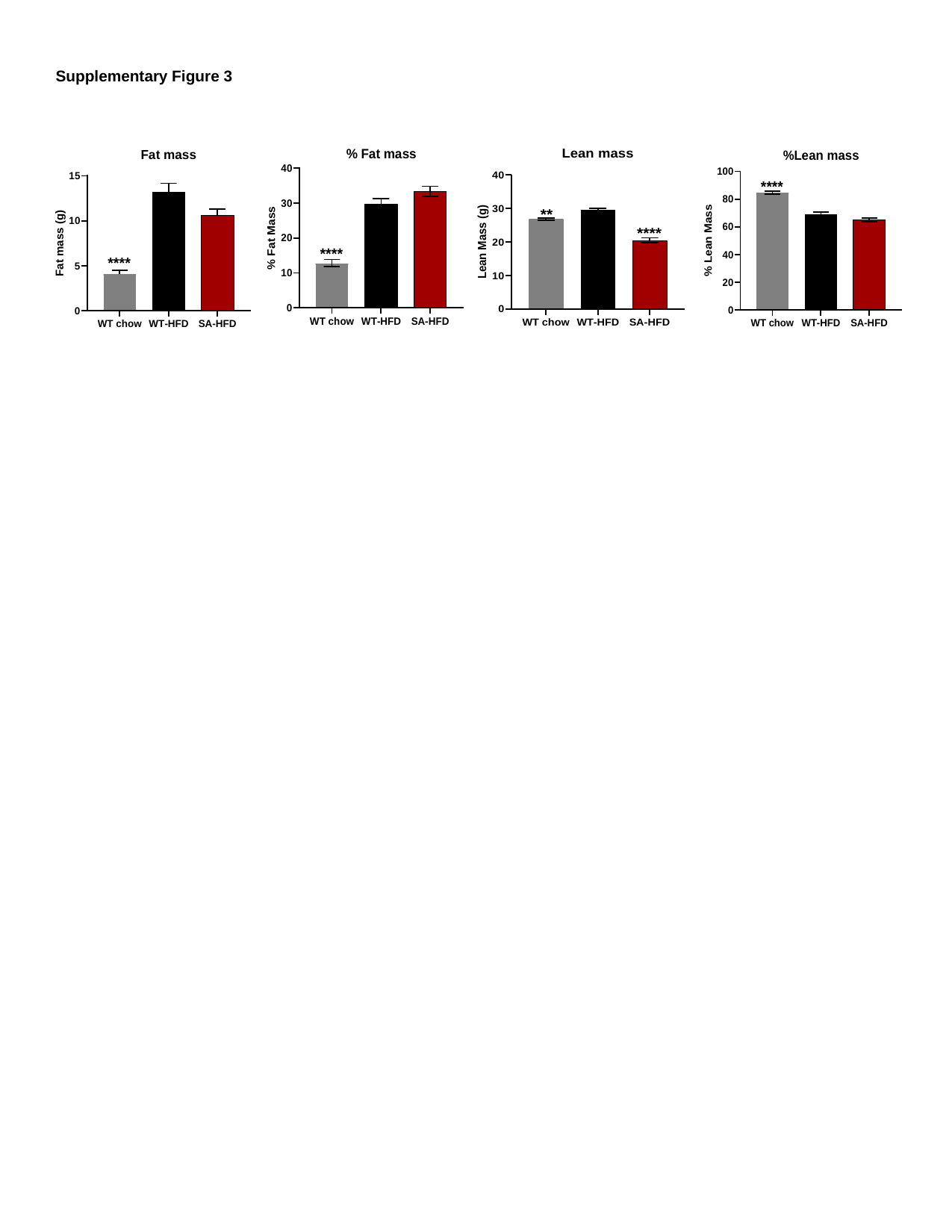

Supplementary Figure 3

## Slide 4
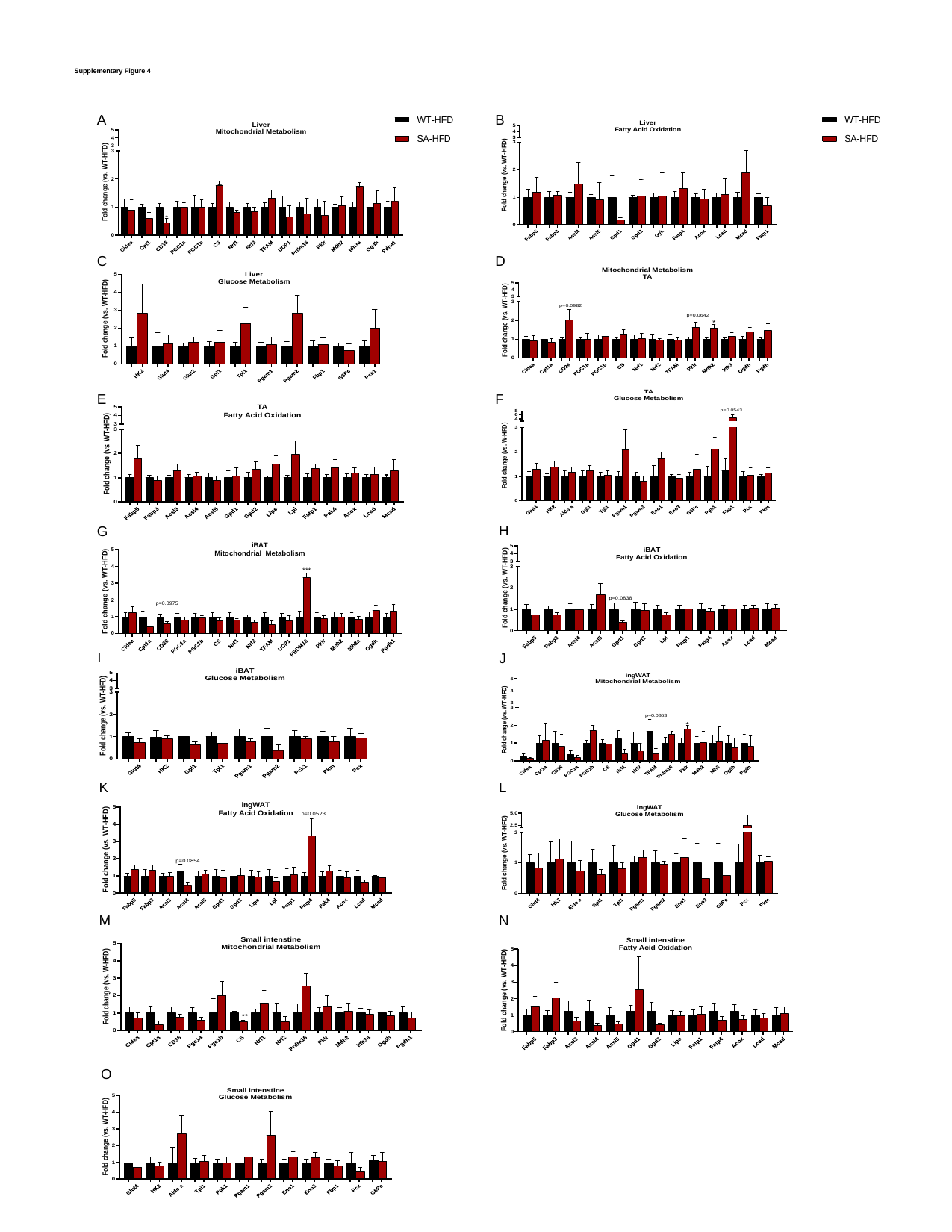

# Supplementary Figure 4
A
B
C
D
E
F
H
G
I
J
K
L
M
N
O

## Slide 5
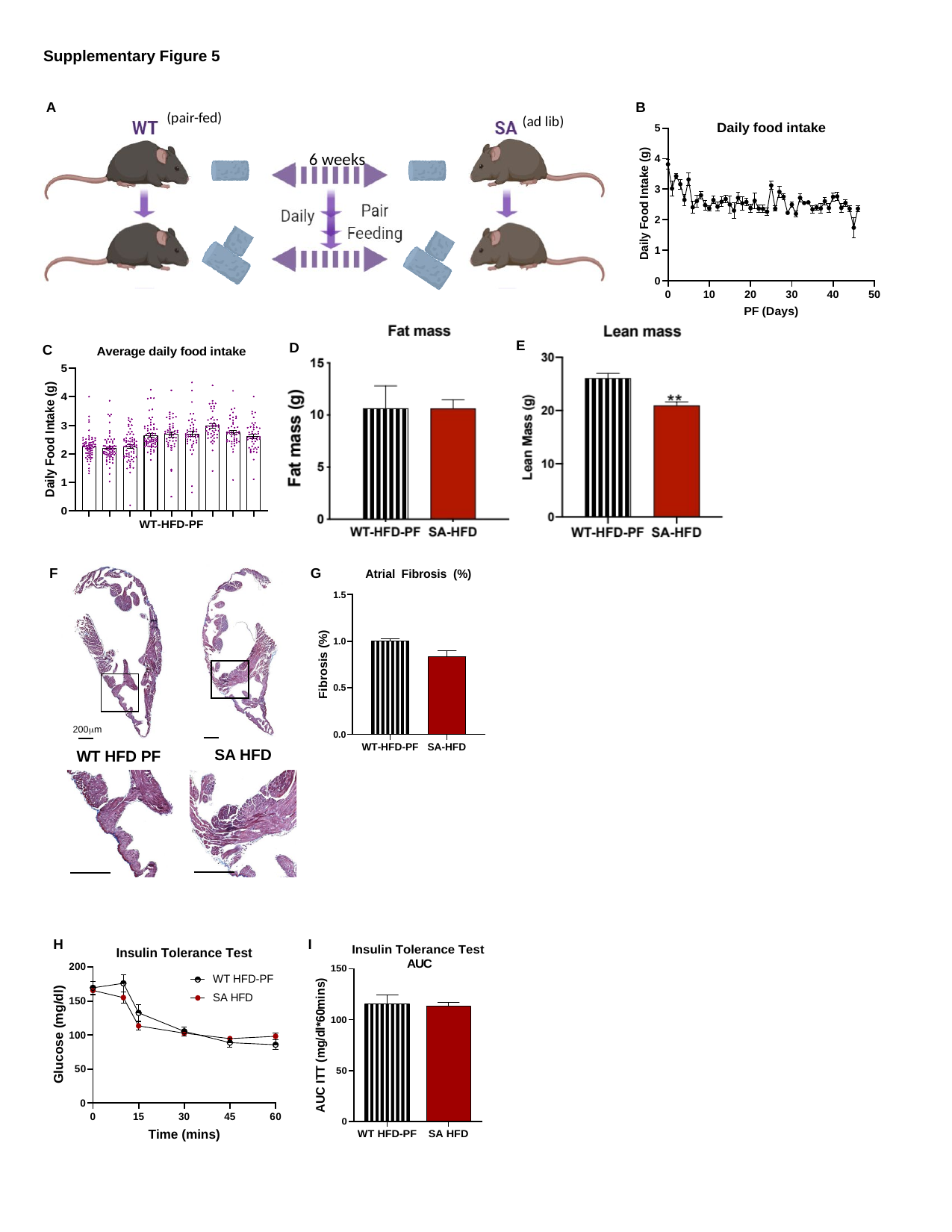

Supplementary Figure 5
A
B
(pair-fed)
(ad lib)
6 weeks
E
D
C
G
F
WT HFD PF
200mm
SA HFD
H
I
